# Mercury biomagnification in the food web of Agami Pond, Kaw-Roura Nature Reserve, French Guiana

**DOI:** 10.1016/j.heliyon.2024.e28859

**Published:** 2024-03-29

**Authors:** Jérémy Lemaire, Rosanna Mangione, Stéphane Caut, Paco Bustamante

**Affiliations:** a Department of Behavioral and Cognitive Biology, University of Vienna, Djerassiplatz 1, 1030, Vienna, Austria; b Consejo Superior de Investigaciones Cientificas (CSIC), Departamento de Etologia y Conservacion de la Biodiversidad, Estacion Biologica de Doñana, C/ Americo Vespucio, s/n (Isla de la Cartuja), E-41092, Sevilla, Spain; c ANIMAVEG Conservation, 58 avenue du Président Salvador Allende, F-94800, Villejuif, France; d Littoral Environnement et Sociétés (LIENSs), UMR 7266 CNRS-La Rochelle Université, 2 rue Olympe de Gouges, 17000, La Rochelle, France

**Keywords:** Freshwater ecosystems, Trace elements, Trophic chain, Agami heron, Black caiman, Top predators

## Abstract

Freshwater ecosystems are among the most important ecosystems worldwide, however, over the last centuries, anthropogenic pressures have had catastrophic effects on them. Mercury (Hg) is one of the main environmental contaminants which globally affect ecosystems and particularly freshwater wildlife. While Hg originates from natural sources, anthropogenic activities such as agriculture, biomass combustion, and gold mining increase its concentrations.

Gold mining activities are the main drivers of Hg emission in tropical ecosystems and are responsible for up to 38% of global emissions. Once in its methylated form (MeHg), mercury biomagnifies through the trophic chain and accumulates in top predators. Due to the toxicity of MeHg, long-lived predators are even more subjected to chronic effects as they accumulate Hg over time.

In the present study we quantified Hg contamination in two top predators, the Black caiman *Melanosuchus niger* and the Agami heron *Agamia agami*, and in their prey in the Kaw-Roura Nature Reserve in French Guiana and evaluated the biomagnification rate in the trophic chain.

Our results show that despite a TMF in the range of others in the region (4.38 in our study), top predators of the ecosystem present elevated concentrations of Hg. We have found elevated Hg concentrations in the blood of adult Black caiman (2.10 ± 0.652 μg g^−1^ dw) and chicks of Agami heron (1.089 ± 0.406 μg g^−1^ dw). These findings highlight the need to better evaluate the potential impact of Hg in freshwater top predators, especially regarding reprotoxic effects.

## 1 Introduction

Freshwater ecosystems are among the most important ecosystems worldwide as they host more than 10% of the world's fauna and one third of all vertebrates [1], and provide valuable ecosystem services [2,3]. However, freshwater ecosystems have been profoundly altered over the last centuries due to anthropogenic pressures such as urbanization, extraction of resources, and agriculture, leading to direct habitat destruction, loss of ecosystem services, and environmental pollution [4,5].

Among the main pollutants, mercury (Hg) is worrying as it is a pervasive contaminant which is found in all ecosystems worldwide. Mercury originates from natural geological sources and anthropogenic activities [6], where artisanal small scale gold mining (ASGM) represents the major anthropogenic source of Hg with 38% of global Hg emissions, and up to 80% in tropical ecosystems [7,8]. In anoxic conditions, inorganic Hg is methylated by microorganisms into methylmercury (MeHg), its most bioavailable and toxic form [9,10]. MeHg biomagnifies through the food chain and accumulates in tissues of top predators, making them particularly vulnerable to its toxic effects [11]. The rate of Hg biomagnification in ecosystems is linked to the latitude of these ecosystems, physico-chemical parameters, and the pool of Hg present in the ecosystem [12]. In general, tropical ecosystems have lower biomagnification factors than temperate ecosystems [12], however, several studies have showed that Hg concentrations in top predators in tropical regions are high [[13], [14], [15], [16], [17], [18]].

Mercury toxicity encompasses a large variety of deleterious effects on human and wildlife health. In 1965, Hg toxicity was revealed due to human poisoning through fish consumption contaminated by MeHg, later called the Minamata disease, a major disaster in human history. Further, Hg toxicity on wildlife and the environment has been demonstrated many times and it is well admitted that environmental Hg contamination represents a global threat for ecosystems and human welfare worldwide [11,19]. Many countries took steps to monitor and regulate Hg and finally, scientific evidence of the toxicity of this metal for the environment led to the Minamata Convention, which aims to reduce the use and release of Hg into the environment [20,21]. In 2013, around 140 countries have approved the Minamata Convention on Mercury. In wildlife, Hg is known to disrupt endocrine regulation, brain function, growth, normal cellular function, physiology, reproduction, and behaviour [[22], [23], [24], [25]]. Long-lived predators are even more subjected to chronic Hg effects as they bioaccumulate the contamination they are exposed to over several decades. However, chronic effects of Hg contamination in tropical species remain understudied.

French Guiana's Kaw-Roura Nature Reserve, a vast wetland ecosystem, hosts two emblematic top predators: one of the biggest remaining populations of Black caiman *Melanosuchus niger*, and the second one being the biggest breeding population of Agami heron *Agamia agami* in French Guiana [26]. However, information on Hg contaminations in these two top predators and their know prey is poor. To the best of our knowledge, there is only one recent study on *Melanosuchus niger* in the Guiana Shield, which has revealed the highest Hg concentrations that have so far been documented in South American caimans [27]. Such elevated Hg concentrations represent a major threat for both Black caiman and Agami heron populations, herons being also prey of caimans. Additionally, natural Hg richness of soil, and intensive gold mining activities in French Guiana are increasing pressure the contamination of this particular ecosystem.

The goals of our study are first to quantify the Hg contamination of these two top predators and their prey and second, to evaluate the biomagnification rate of the trophic chain to better understand the risk related to Hg contamination in this ecosystem.

## 2 Material and methods

### 2.1 Sampling

**Fig. 1:**
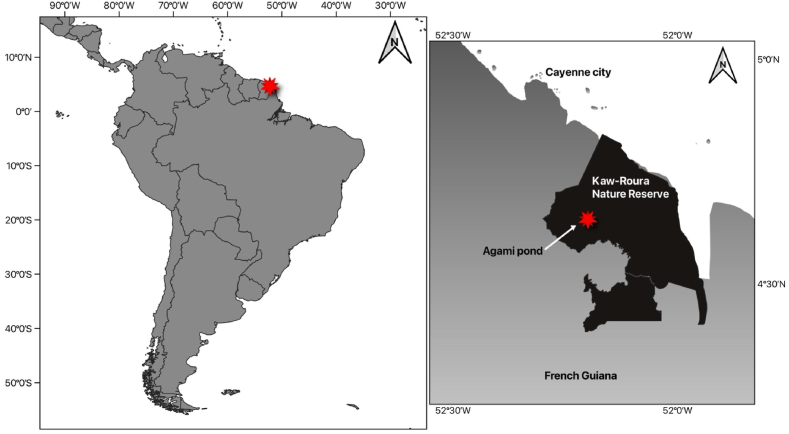
Location of Agami Pond in the Kaw-Roura nature reserve, French Guiana.

The study was carried out in the Kaw-Roura Nature Reserve in French Guiana, a vast marsh dominated by floating vegetation, located 40 km southeast of Cayenne (Fig. 1). Sampling was performed in one of the permanent open water areas, namely Agami Pond (04° 38′ N, 52° 09’ W), which is equipped with a floating research platform (6 × 4m). One of the particularities of the area is that Agami Pond is inhabited by the biggest known population of Black caiman in French Guiana and additionally hosts a large breeding colony of Agami herons during the rainy season.

Sampling of the food web was initially carried out as part of another study [28] and included 12 different species, from plankton to adult caimans (Table 1). Briefly, Black caimans were captured at night, and for each individual the body length was determined (total length) and whole blood was collected from the cranial sinus using a syringe with a 30-gauge heparinized needle. Newly hatched Agami heron chicks were taken directly from their arboreal nests and three drops of whole blood from the femoral vein were collected using heparinized capillary tube. Fishes were collected using nets and rods, then we took a sample of dorsal muscle for large species and the entire individual for smaller species.

**Table 1:** Mercury concentration (μg.g^−1^ dw), ***δ***^15^N and ***δ***^13^C (‰) values, and trophic level (TL) in different tissues of the trophic chain of Agami Pond, Kaw-Roura Nature Reserve, French Guiana.

Species	Tissue	n	Hg (μg.g^−1^)	***δ***^15^N (‰)	***δ***^13^C (‰)	TL
Zooplankton	–	–	–	0.49 ± 0.41	−30.30 ± 0.33	
**Crustaceans**						
*Macrobrachium jelskii*	Muscle	9	0.033 ± 0.010	6.12 ± 0.31	−29.56 ± 0.38	3.65 ± 0.09
**Amphibians**						
*Pipa snethlagea*	Muscle	4	0.162 ± 0.027	8.39 ± 0.46	−28.97 ± 0.59	4.32 ± 0.13
**Omnivorous fishes**						
*Metynnis lippincottianus*	Muscle	2	0.068 ± 0.021	3.41 ± 1.04	−32.62 ± 1.46	2.86 ± 0.30
*Hemigrammus* sp.	Muscle	6	0.240 ± 0.133	4.87 ± 0.28	−32.81 ± 2.20	3.29 ± 0.08
*Pristella maxillaris*	Whole body	9	0.198 ± 0.063	5.38 ± 0.72	−31.30 ± 2.21	3.44 ± 0.21
*Chaetobranchus flavescens*	Muscle	5	0.265 ± 0.168	6.46 ± 0.28	−28.58 ± 0.76	3.76 ± 0.09
**Carnivorous fishes**						
*Crenicichla saxatilis*	Muscle	1	0.264	6.05	−30.83	3.64
*Hoplerythrinus unitaeniatus*	Muscle	4	1.614 ± 1.093	8.14 ± 1.15	−27.07 ± 1.19	4.25 ± 0.34
*Hoplias malabaricus*	Muscle	5	1.429 ± 0.688	9.12 ± 0.72	−27.35 ± 0.54	4.54 ± 0.21
**Birds**						
*Agami agamia*	Blood	24	1.089 ± 0.406	7.79 ± 0.60	−28.95 ± 0.70	4.15 ± 0.18
**Caimans**						
*Melanosuchus niger* (category A)	Blood	2	0.365 ± 0.092	4.48 ± 0.13	−27.32 ± 0.67	3.17 ± 0.04
*Melanosuchus niger* (category B)	Blood	27	0.784 ± 0.467	5.56 ± 0.94	−27.60 ± 0.67	3.49 ± 0.28
*Melanosuchus niger* (category C)	Blood	31	1.464 ± 0.376	7.13 ± 0.34	−27.49 ± 0.67	3.95 ± 0.10
Melanosuchus niger (category D)	Blood	12	2.100 ± 0.652	7.34 ± 0.30	−27.47 ± 0.77	4.05 ± 0.09

### 2.2 Mercury analysis

All samples (including whole blood, muscle and whole individual) were freeze-dried for 48h, ground in powder and homogenized. Total Hg was determined using an atomic absorption spectrometer AMA-254 (Advanced Mercury Analyser-254; Altec®). Analyses were made on at least two replicates of 0.8–8.0 mg dry weight (dw) for each individual. The reproducibility for duplicate samples was approved when Relative Standard Deviation (RSD) was below 10%. At the beginning and at the end of each analytical cycle, and every 10 samples, an analysis of certified reference material TORT-3 (Lobster hepatopancreas from the National Research Council of Canada; certified Hg concentration: 0.292 ± 0.022 μg g^−1^ dw) was performed to validate the method. Measured values for TORT-3 were 0.292 ± 0.002 μg g^−1^ (n = 20), giving a recovery of 100.5 ± 2.1 %. Blanks were included at the beginning of each analytical run and the limit of quantification of the AMA was 0.05 ng. Hg concentrations are further expressed in μg.g^−1^ dw. Hg concentrations in zooplankton were not determined due to insufficient material to carry out the analyses.

### 2.3 Isotope analysis

An analysis of nitrogen and carbon stable isotopes was conducted on samples after being freeze-dried and then grounded to a fine powder. Aliquots of 0.3–0.4 mg were placed in tin capsules. Stable isotopes were analysed using a mass spectrometer (IsoPrime 100, Isoprime, UK) associated to C–N–S elementary analyser (Vario MICRO cube, Elementar, Germany). Stable carbon and nitrogen isotope ratios are expressed as (***δ***^15^N) or $\left(\delta^{13}C\right)=\left[\left(\frac{R_{sample}}{R_{standard}}\right)-1\right]*1000$, where R is ^15^N/^14^N or ^13^C/^12^C for ***δ***^15^N or ***δ***^13^C. The replicate assays of internal laboratory standards indicated maximum measurement errors of ± 0.2 ‰ for both the nitrogen and carbon isotope measurements (n = 10).

### 2.4 Statistical analysis

All analyses were performed using the software R, v.4.2.2 (*R development Core Team*). The normality and the homogeneity of variance were first checked, and data were log (natural)-transformed for Hg concentration. *Melanosuchus niger* data were separated in four different groups depending on total length of animals, such as neonates < 50 cm (category A), 50 < juveniles < 120 cm (category B), 120 cm < subadults < 200 cm (category C), and adults > 200 cm (category D), due to the shift in the species’ trophic ecology [28]. Differences in Hg concentrations (Log[Hg]) between species were assessed by ANOVA. Relationship between Hg concentrations (Log[Hg]) and the ***δ***^15^N values was assessed by linear regression.

The trophic level (TL) of each species was calculated according to the equation: ${TL}_{consumer}=\left(\delta^{15}N_{consumer}-\;\delta^{15}N_{baseline}\right)/\;\Delta^{15}N+\lambda$.

where ***δ***^15^N_consumer_ and ***δ***^15^N_baseline_ are the isotope values of consumers and the lowest ***δ***^15^N of the sampled species (baseline), i.e., zooplankton as primary consumer (trophic level 2). λ is the trophic level of the organism used as ***δ***^15^N_baseline_ (=2), and $\Delta^{15}N$ as the trophic enrichment factor in the food web, established at 3.4 ‰ [12].

The Trophic Magnification Slope (TMS) value was obtained using the slope (*b*) of the relationship between Hg concentrations (Log[Hg]) and ***δ***^15^N values [12].

The Trophic Magnification Factor (TMF) was calculated following the equation: TMF = $10^{\left(b\;*\;3.4\;‰\right)}$, where *b* is the slope of the linear regression between Hg concentrations (Log[Hg]) and the ***δ***^15^N values, and 3.4 ‰ is an average increase in ***δ***^15^N with each trophic level [12]. The TMF provides a mean rate of increase per trophic level in the studied food web and assumes that uptake is directly related to the diet as the main exposure route [29], where TMF > 1 indicates Hg biomagnification.

Relationships between Hg concentrations (Log[Hg]) and TL were assessed by linear regression.

## 3 Results

Mercury has been detected in the tissues of all specimen that were sampled and analysed as part of this study, with the exception of zooplankton, for which only isotopic analysis has been performed. Mercury concentrations were highly variable between species (ANOVA: F_13,121_ = 47.17, p < 0.001; Table 1), with the lowest Hg concentration in the muscle of the shrimp *Macrobrachium jelskii* (Palaemonidae) (0.033 ± 0.010 μg g^−1^ dw) and the highest Hg concentration in blood of adult Black caiman *Melanosuchus niger* (2.10 ± 0.652 μg g^−1^ dw).

Values of ***δ***^15^N ranged from 0.49 ± 0.41 ‰ for zooplankton to 9.12 ± 0.72 ‰ for the wolf fish *Hoplias malabaricus*, while ***δ***^13^C values ranged from −32.81 ± 2.20 ‰ for the tetra *Hemmigrammus* sp. to −27.07 ± 1.19 ‰ for the aimara *Hoplerythrinus*.

Results showed a positive relationship between Hg concentrations and ***δ***^15^N values (Linear regression: slope = 0.11 ± 0.09, R^2^ = 0.266, p < 0.001). Based on the ***δ***^15^N value for zooplankton (i.e., 0.49 ± 0.41 ‰), which has a TL of 2 as a primary consumer, the TLs of the other species were determined. The calculated TLs are given in Table 1.

The lowest TL was 2.86 ± 0.30 for the spotted silver dollar, *Metynnis lippincottianus* (Serrasalmidae), the highest TL 4.54 ± 0.21 for the wolf fish *Hoplias malabaricus* (Erythrinidae).

**Fig. 2:**
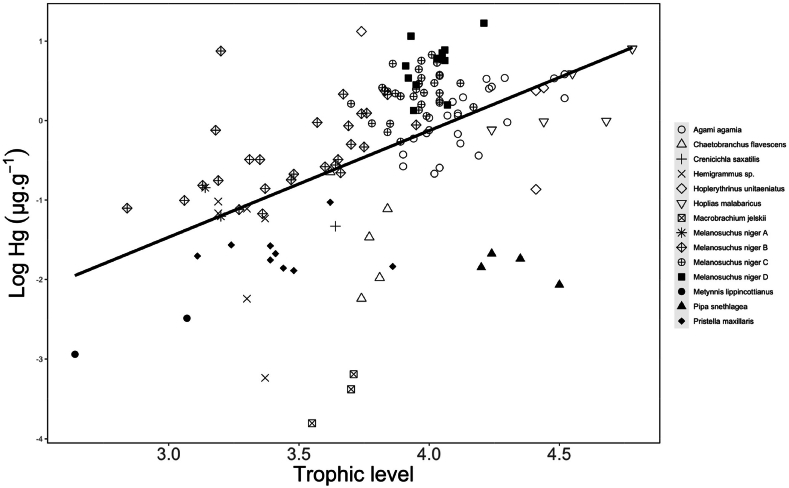
Linear regression between Log of Hg concentration (μg.g^−1^ dw) and trophic level (TL) in the trophic chain of Agami Pond, Kaw-Roura Nature Reserve, French Guiana. Linear regression based on ***δ***^15^N (TMS): slope = 0.11 ± 0.09, R^2^ = 0.266, p < 0.001) and linear regression based on trophic level (TMF): slope = 0.19 ± 0.02, R^2^ = 0.266, p < 0.001.

TMS obtained via linear regression between the logarithm of Hg concentrations and the ***δ***^15^N values was 0.11. Calculation from TMS has showed that TMF of Hg was 4.38 in the studied trophic food web (TMF: slope = 0.19 ± 0.02, R^2^ = 0.266, p < 0.001, Fig. 2).

## 4 Discussion

Mercury contamination in French Guiana's freshwater ecosystems has been extensively studied in fishes [14,[30], [31], [32], [33]], mainly because fish represent a major source of exposure which poses a threat on local communities. In contrast, quantification of Hg concentrations and biomagnification and the assessment of impacts on top predators, such as birds and reptiles, remain limited to few studies [[34], [35], [36]]. The present study site, Agami Pond, is home to two emblematic top predators of major conservation interest, the Agami heron *Agami agamia* and the Black caiman *Melanosuchus niger*, which underlines the importance to this marsh ecosystem. Because Hg is highly methylated in the aquatic environment, these predators, which are positioned on top of the food chain, are expected to have high Hg concentrations and to consequently suffer from its deleterious effects.

The trophic magnification factor of Hg which we have found in Agami Pond (TMF = 4.38) is in the range of TMFs found in other freshwater ecosystems in French Guiana (e.g., “Petit Saut” TMF = 4.48, and “Sinnamary River” TMF = 4.38 [37]) and is in accordance with the global TMF in freshwater ecosystems (global TMF = 4.43 ± 4.48 [12]). However, the TMF was higher than African subtropical wetland (TMF = 2.7) [38]. Our results show that biomagnification of Hg occurring in Agami Pond is within a normal range when compared with other studies carried out in the region, and worldwide. However, it is important to acknowledge that due to permit restrictions when working in a nature reserve, the recommendation of three TLs to calculate the TMF was not reached and could limit the obtained results.

Mercury contamination in South America originates from natural sources, atmospheric deposition, and anthropogenic activities such as gold mining [39,40]. In French Guiana, forest soils are known to have relatively high natural concentrations of Hg (mean of 0.3 μg g^−1^ dw [41]), which are increased by atmospheric deposition [42,43], and local gold mining activities [44]. At our study site, Hg contamination seems to be mostly related to soil erosion from the mountain range “Montagne de Kaw” and high atmospheric deposition in the region [12,45], as recent gold mining activities are not present in this watershed. The Hg content observed in the trophic chain of this particular ecosystem cannot be attributed to specific human activities.

In our study, the shrimp *Macrobrachium jelskii* has the lowest Hg concentrations with 0.033 ± 0.010 μg g^−1^ dw. This value is in accordance with the Hg concentrations found in the shrimp *Macrobrachium amazonicum* (0.033 ± 0.001 μg g^−1^ dw) in a natural environment in Brazil [46]. In fishes, carnivorous species show the highest Hg concentrations with respectively 1.429 ± 0.688 μg g^−1^ dw for the wolf fish *Hoplias malabaricus,* and 1.614 ± 1.093 μg g^−1^ dw for the aimara *Hoplerythrinus unitaeniatus*. These Hg concentrations are in agreement with those reported in other publications on these two species in French Guiana [33,34]. Although Hg concentrations we have found in fish are not the highest documented in French Guiana, even low Hg concentrations can have adverse effects on behaviour and reproduction, and can cause organ alteration and cellular injury, as numerous studies have shown [[47], [48], [49], [50]], therefore needing further investigation.

As a piscivorous bird, *Agamia agami* shows relatively high blood Hg concentrations (1.089 ± 0.406 μg g^−1^ dw) compared to other freshwater birds from the Brazilian Amazon region, where average blood values in adults and chicks range between 0.65 ± 0.29 μg g^−1^ dw in the neotropical cormorant *Nannopterum brasilianus* to 1.87 ± 2.30 μg g^−1^ dw in the anhinga *Anhinga anhinga* [51]. The Hg concentrations we have found in chicks of *Agamia agami* are worrying but are not exceed the threshold for Hg toxicity in birds (blood Hg concentration 9.6 μg g^−1^ dw., converted from ww. to dw., using moisture content of 79,13% [52]), for which an impact on physiology, reproduction, and behaviour has been observed [53,54]. The potential reprotoxic effect of such Hg contamination in this large breeding population needs to be better understood, especially as *Agamia agami* is classified as vulnerable by the IUCN Red List of Threatened Species [55].

Despite our expectations, results have showed that *Melanosuchus niger* does not have the highest trophic level, though the highest Hg values were found in adults individuals (category D) with 2.100 ± 0.652 μg g^−1^ dw, while juveniles show low values of 0.365 ± 0.092 μg g^−1^ dw. To date, these values are the highest reported in a South American caiman species. Hg quantified in blood of vertebrates represents a proxy of MeHg (>80% [56,57]), the most toxic form of Hg, concentrations found in the species can have drastic effects and therefore represent a serious threat. It has already been shown that low levels of Hg in other caiman species from the same region can affect physiology (0.676 ± 0.414 μg g^−1^ dw [37]), which ultimately stresses the importance of special attention on this Black caiman population.

In tropical ecosystems, efficiency of Hg trophic transfer is reduced at each trophic level, thus reducing biomagnification [12]. The present high concentrations of Hg in adult caimans can potentially be explained by the particularity of the Agami Pond. At this site, large caimans feed on Agami herons during their breeding season, which increases their Hg contamination. Selenium (Se) has protective properties against Hg toxicity in organisms [58,59], however, blood Se concentrations in the *Melanosuchus niger* population in Agami Pond are low [60]. These results highlight the need to assess Hg and Se in the Agami Pond food web in order to evaluate the potential impact of Hg contamination on this particular ecosystem.

Hg concentrations found in the Agami Pond food web suggest that the bioavailable, environmental concentrations of Hg remain high despite the absence of direct anthropogenic activities that could be responsible for this contamination. A comparative study on museum specimen from Kaw-Roura Nature Reserve to assess the evolution of Hg contamination in this particular ecosystem would be highly interesting. In addition, the levels of Hg found in the trophic chain, and particularly in *Agamia agami* and *Melanosuchus niger,* deserve further studies to assess potential long-term adverse effects of Hg contamination in these two long-lived species, particularly regarding the reprotoxic effect of this contaminant.

## Data availability statement

Data associated to the study is not deposited into a publicly available repository, however, data will be made available upon request.

## CRediT authorship contribution statement

**Jérémy Lemaire:** Writing – review & editing, Writing – original draft, Funding acquisition, Formal analysis, Conceptualization. **Rosanna Mangione:** Writing – review & editing, Writing – original draft. **Stéphane Caut:** Investigation. **Paco Bustamante:** Writing – review & editing, Writing – original draft.

## Declaration of competing interest

The authors declare that they have no known competing financial interests or personal relationships that could have appeared to influence the work reported in this paper.
